# Selection for female traits of high fertility affects male reproductive performance and alters the testicular transcriptional profile

**DOI:** 10.1186/s12864-017-4288-z

**Published:** 2017-11-21

**Authors:** Marten Michaelis, Alexander Sobczak, Dirk Koczan, Martina Langhammer, Norbert Reinsch, Jennifer Schoen, Joachim M. Weitzel

**Affiliations:** 1 Institute of Reproductive Biology, University of Rostock, Rostock, Germany; 2 Institute of Genetics and Biometry, Leibniz Institute for Farm Animal Biology (FBN), Dummerstorf, Germany; 3 Institute of Immunology, University of Rostock, Rostock, Germany; 4 Leibniz Institute for Farm Animal Biology (FBN), Institute of Reproductive Biology, FBN Dummerstorf, Wilhelm-Stahl-Allee 2, 18196, Dummerstorf, Germany

**Keywords:** Long-term selection mouse lines, Outbred mouse model, High-fertility, Fecundity, Testis, Reproductive fitness, Sperm motility, Casa

## Abstract

**Background:**

Many genes important for reproductive performance are shared by both sexes. However, fecundity indices are primarily based on female parameters such as litter size. We examined a fertility mouse line (FL2), which has a considerably increased number of offspring and a total litter weight of 180% compared to a randomly bred control line (Ctrl) after more than 170 generations of breeding. In the present study, we investigated whether there might be a parallel evolution in males after more than 40 years of breeding in this outbred mouse model.

**Results:**

Males of the fertility mouse line FL2 showed reduced sperm motility performance in a 5 h thermal stress experiment and reduced birth rate in the outbred mouse line. Transcriptional analysis of the FL2 testis showed the differential expression of genes associated with steroid metabolic processes (Cyp1b1, Cyp19a1, Hsd3b6, and Cyp21a1) and female fecundity (Gdf9), accompanied by 150% elevated serum progesterone levels in the FL2 males. Cluster analysis revealed the downregulation of genes of the kallikrein-related peptidases (KLK) cluster located on chromosome 7 in addition to alterations in gene expression with serine peptidase activity, e.g., angiotensinogen (Agt), of the renin-angiotensin system essential for ovulation. Although a majority of functional annotations map to female reproduction and ovulation, these genes are differentially expressed in FL2 testis.

**Conclusions:**

These data indicate that selection for primary female traits of increased litter size not only affects sperm characteristics but also manifests as transcriptional alterations of the male side likely with direct long-term consequences for the reproductive performance of the mouse line.

**Electronic supplementary material:**

The online version of this article (10.1186/s12864-017-4288-z) contains supplementary material, which is available to authorized users.

## Background

Fertility is complex, involving numerous interactions of different pathways. Most knowledge concerning the interaction of genes has been obtained from exploratory models, such as transgenic or knockout mice. The database Mouse Genome Informatics (MGI - www.informatics.jax.org) harbors more than 2000 genotypes associated with reproductive phenotypes. Although there are cases of duplicated annotation, nearly all of these annotations refer to an infertile or at least subfertile phenotype. Only a minority (<1%) of annotations have been associated with enhanced fertility or improved reproductive performance (see Table 1). This correlation is clear evidence for transgenic alterations resulting in a decreased fertility phenotype. Nevertheless, genetically interventional studies are unquestionably helpful in dissecting fertility-relevant pathways. The above comparison argues for a shortage of models of increased reproductive performance to understand this condition. In addition, a majority of transgenic models are monogenetic, despite the common view that (in)fertility is multi-causal, and a network of genes is essential for reproductive processes. As such, we expect not only a single gene but also a combination of genes to be reproductively responsive.

**Table 1:** Genotypes associated with a certain fertility phenotype, its amount and proportion [%] on total phenotype number. Data are extracted (May, 2016) from the database ‘Mouse Genome Informatics’ (http://www.informatics.jax.org/vocab/mp_ontology/MP:0002161)

Mammalian phenotype *(number of matching genotypes)*	
Abnormal fertility/fecundity *(2479 genotypes)*	total
Infertility (*1462 genotypes*)	59%
Reduced fertility (*888 genotypes*)	36%
Enhanced fertility (*4 genotypes*)	0.2%
Decreased litter size (*441 genotypes*)	18%
Increased litter size (*25 genotypes*)	1%

We report on a unique murine animal model selected for the ‘high-fertility’ phenotype over more than 170 generations for use as an alternative experimental strategy for overcoming the above-mentioned limitations. The additional advantage of this approach is its heterogeneity, which more closely mimics the phenotypic alterations in nature compared to single gene approaches to generate classical transgenic or knockout models.

In the 1970s, the Leibniz Institute for Farm Animal Biology (FBN) established two mouse models for high reproductive performance through long-term selective breeding: fertility lines 1 and 2 (FL1 and FL2). The improved fertility selection criteria included (i) number of offspring and (ii) total litter birth weight at first delivery. Both selection criteria have been combined in a breeding index = 1.6× litter size + litter birth weight. The selection for litter size and litter weight via breeding index avoided intrauterine growth retardation of the offspring. The factor 1.6 reflects the average birth weight of single pups. Animals of the largest and heaviest litters were recruited for breeding of the next generation. In parallel, an unselected control line (Ctrl) was developed based on the same initial founder population. All specifications regarding breeding and the proceedings of these mouse lines have been reviewed [1–3]. Until recently, these fertility lines have lacked a profound molecular biological description, and to fill this gap, we exclusively focused on FL2 characterization in the present study.

Based on previous studies, FL2 females ovulate more oocytes and harbor almost twice as many corpora lutea (CL) as Ctrl females [4]. The intrauterine growth and development of embryos was similar between both mouse lines [4]. Thus, the increased ovulation occurs in response to selection pressures. Moreover, FL2 females showed an increase in serum progesterone (P4) levels over the estrus cycle [4]. On the male side, FL2 bucks showed decreased lifetime expectancy and a more explorative behavior in an open field test [2, 5]. This result indicates that breeding focused on female high reproductive performance not only affects the females but also the males. Despite selection criteria exclusively visible on the dam side, we examined the physiological and genetic responses of the male side to selection for high female fecundity traits after more than 170 generations of breeding.

Many genes essential for reproductive performance are shared within the germinal organs of both sexes. Thus, we hypothesized that during the female-focused selection for high litter size, achieved through the increased ovulation rate in FL2 females, the male side is also affected, particularly in the male germinal organ.

The main task of the testis is the production of functional gametes; hence, we analyzed sperm motility as a physiological parameter for overall testicular function. Furthermore, we compared FL2 and Ctrl testicular transcriptional profiles on a global gene expression level.

## Methods

### Animal model & ethics statement

The animal experiments have been approved by the local authorities (Landesamt für Landwirtschaft, Lebensmittelsicherheit und Fischerei, Mecklenburg-Vorpommern, Germany). The mouse lines were maintained in a specific pathogen-free (since 2012) environment with a 12:12 h light-dark regime and ad libitum access to water and food (ssniff® M-Z, Soest, Germany) at the laboratory animal facility of the FBN.

All mouse lines were originally derived from the same genetic pool of a mixture of eight defined founder mouse lines. From this starting population, the high-fertility line FL2 was generated during a long-term selection experiment for more than 40 years referring to >170 generations. The selection was performed via breeding index, combining first litter size and total litter birth weight (Dummerstorf breeding index: 1.6× litter size + litter birth weight). Offspring of litters with the highest breeding indices were recruited to breed the next generation. Until the 23rd generation, the FL2 females were cycle synchronized using gestagen chlormadinone acetate. In parallel, an unselected Ctrl line was generated from the same starting population and has been maintained under identical housing conditions using a rotational mating scheme and avoiding full sib mating to decrease the average rate of inbreeding. To ensure the outbred character, the mouse lines were bred with a population size of 60–100 and 125–200 animals per generation for FL2 and Ctrl lines, respectively. However, reflecting the breeding process, the corresponding inbreeding values accounted for 0.175 for the Ctrl and 0.977 for the FL2 lines [6]. Details regarding the breeding procedures can be found in Dietl et al. and Schüler et al. [1–3]. The males of this investigation have been further described in a two-factorial breeding experiment to delineate the impact of males and females on fertility parameters [6]. Birth rates (accounted as: deliveries (living and nonliving litters) per mating) were extracted from standard breeding data acquired over a 4-year period and encompassing at least 840 pairings per line (14 generations with at least 60 breeding pairs in FL2 and 125 pairs in Ctrl). Statistical analysis was performed using Student’s t-test.

### Sperm motility analysis - sperm motility stress test

The sperm motility analysis was accomplished using CASA (computer-assisted sperm analysis) via SpermVision (Minitube, Germany). Prior to a 5 h thermal stress procedure and CASA measurements, 12-week-old males (*n* = 10 per line) were euthanized by CO_2_ inhalation. To obtain sperm, the cauda epididymis was extracted, cleaned and minced (five cuts) in freshly prepared 300 μl of spermatozoa-suitable M199 media (M7528, Sigma-Aldrich, Germany) and incubated for 5 min at 37 °C for sperm release. Tissue remnants were subsequently filtered using 30-μm mesh.

To generate stress conditions, the spermatozoa suspension was continuously exposed to 37 °C for a 5-h thermal stress period. Every hour, the current sperm motility characteristics were determined by quickly applying a 3-μl aliquot to 37 °C tempered chamber slides (20 μm, Leja, Netherlands). Each sperm sample was considered as the average of 8 defined chamber partitions viewed and measured using the CASA system (Minitube, Germany).

### RNA extraction

For microarray and qPCR studies, the testes of 12–13-week-old males were dissected and snap frozen in liquid N2 or preserved in RNAlater® (Ambion, Austin, USA). According to the manufacturer’s instructions, total RNA was isolated using the RNeasy® Mini Kit (Qiagen, Germany) or the InviTrap Spin RNA Mini Kit (Stratec, Germany) with the simultaneous removal of genomic DNA traces. RNA integrity and quantity were assessed using capillary electrophoresis (Agilent 2100 Bioanalyzer, Santa Clara, USA).

### Sample labeling and hybridization of microarray

For transcriptome profiling, we used the GeneChip® Mouse Transcriptome Array (MTA) 1.0 (released in May, 2015; currently termed Clariom™ D assay, Affymetrix Inc., Santa Clara, USA) with more than 66,100 coding and non-coding transcripts. We used two separate sets of microarray experiments (denoted as 1st and 2nd array set). The 1st array set was based on 8 testicular samples hybridized to individual gene chips. For validation, a 2nd array set was utilized, comprising 8 biologically independent replicates pooled in equivalent amounts and hybridized to a single ‘pool-microarray’ for each mouse line. RNA labeling and hybridization were conducted at the Core Facility for Microarray Analysis, University of Rostock. Briefly, 200 ng of quality controlled total RNA was used for cDNA preparation and labeling with the GeneChip® WT PLUS Reagent Kit (Affymetrix, Santa Clara, USA). The fragmented (~100 bp) and biotinylated cDNA was hybridized for 16 h at 45 °C to Affymetrix Gene Chip® MTA 1.0, followed by washing and staining using the Affymetrix Fluidics Station 450 according to standard instructions. The chips were subsequently scanned at 0.7-μm resolution (GeneChip Scanner 3000 7G, Affymetrix). Furthermore, all hybridizations were assessed for quality requirements, and raw data were submitted to the Gene Expression Omnibus (GEO) database according to MIAME guideline (GSE86063).

### Microarray data normalization and statistical analysis

Raw data cell intensity files were normalized by the Robust Multiarray Average (RMA) algorithm with a Signal Space Transformation (SST) employing the Affymetrix Expression Console software (EC, version 1.4.1.46). Within the same software package, data were further explored using principal component analysis (PCA). Additionally, unsupervised hierarchical cluster analysis was accomplished using Transcriptome Analysis Console software (TAC, Affymetrix, version 3.0.0.466). The same software package was used to calculate fold-changes (FC) and perform statistical analyses. For gene expression detection, an alternative splicing analysis algorithm in the Affymetrix software packages EC and TAC was applied. As recommended, a transcript was considered expressed when at least 50% of the eligible exons were detected above background (DABG, *p* < 0.05).

For data mining, we employed the web-based Database for Annotation, Visualization and Integrated Discovery (DAVID 6.8, david.ncifcrf.gov) [7, 8] and the PANTHER classification system (www.pantherdb.org) [9, 10].

### Quantitative real-time PCR (RT-qPCR)

Microarray results were additionally confirmed for a selected group set of genes using quantitative real-time PCR (qPCR). To this end, 0.5 μg of total RNA was reverse transcribed using random hexamer primers and the iScript cDNA Synthesis kit (BioRad, Germany) in accordance to the manufacturer’s instructions. Briefly, 2 μl of a 1:5 cDNA dilution were amplified with technical duplicates using a SYBR Green mix (BioRad, Germany) and 40 cycles of 30 s at 95 °C, 45 s at 56 °C, and 30 s at 72 °C, as previously described [5]. The primers were designed using the web-based software Primer-BLAST (www.ncbi.nlm.nih.gov/tools/primer-blast) [11]. When possible, special care was taken to select oligonucleotides binding to intron-spanning exons. In addition, specificity control was assessed using gel electrophoresis. The primers used in the present study are listed in the Additional file 1. The samples were normalized to a combination of reference genes (36B4, GAPDH, HPRT, and B2m) and statistically evaluated using the Relative Expression Software Tool (REST 2009) [12].

### Hormone analysis

Serum progesterone was measured by ^3^H–radioimmunoassay using a [1,2,6,7-3H] progesterone (Hartmann Analytic, Germany) tracer as previously described [13]. Briefly, 50 μl of serum in duplicate (*n* = 14 per group) was analyzed with an incubation step at 37 °C for 30 min and 4 °C for 2 h. The B/F separation was performed by a dextran-charcoal method. The radioactivity was quantified using a Liquid Scintillation Counter with an integrated RIA program (TriCarb 2900 TR; Perkin-Elmer, Waltham, USA). Intra- and interassay precision was 7.6% and 9.8%, respectively, for progesterone. The standard curve ranged from 6.25 to 1600 pg/ml, and the detection limit corresponded to 7 pg/ml for progesterone. Statistical analysis was performed using Student’s t-test.

## Results

### Animal model

Studies in mice almost exclusively focus on a single gene approach using transgenic intervention in gain or loss-of-function analyses. We changed the perspective by investigating a mouse model for the long-term selection of the primary female trait of high-fertility visible in increased litter size. Thus, nature itself has selected the genetic alteration to match the selection criteria, in contrast to the classical transgenic approach.

After 172 generations, the randomly selected Ctrl line showed an average litter size of 11.4 ± 3.3 pups, while the total litter weight accounted for 20.8 ± 4.8 g. The extent of the delivery parameters has basically remained the same during the entire random selection process. In contrast, FL2 almost doubled in litter size (+92%) compared to the Ctrl line.

Similar results were obtained for the selection criterion of total litter birth weight (+94%), explaining why we detected no changes in individual newborn weights. An overview of the breeding status after 40 years, with respect to litter size and litter weight, is summarized in Table 2. The line differences in both traits were highly significant (*p* < 0.0001). The gain in reproductive performance was accompanied by no impairment of pub survival. Indeed, intrauterine growth and development were similar between lines, and the body weight of the individual newborns was not reduced in FL2 compared to the Ctrl line [4].

**Table 2:** Number of offspring per litter and total litter weight at birth for Ctrl and FL2 mouse lines after 172 generations of selection

Mouse line	Ctrl	FL2
Offspring per litter	11.4 ± 3.3	21.9*** ± 2.6 (192%)
Total litter weight at birth [g]	20.8 ± 4.8	40.4*** ± 4.5 (194%)

Proportions (%) to Ctrl are indicated. Data were tested for normal distribution and analyzed by Student’s t-test (***, *p* < .0001)

Previous studies have demonstrated that the selection process is associated with increased ovulation and alterations in germinal organs of FL2 females [4, 14]. However, molecular and functional information concerning whether FL2 male germinal organs are affected by the selection process was lacking. To address this issue, we examined sperm motility parameters using CASA and evaluated the overall birth rate of the mouse line acquired over a 4-year breeding period. In addition, we performed a gene expression analysis of the central male reproductive organs, the testis.

### Sperm motility and birth rate

Computer-assisted sperm motility analysis (CASA) was conducted to characterize the males at physiological and functional levels, focusing on the quality of the sperm of FL2 and Ctrl males. To this end, spermatozoa of the cauda epididymis were released into media. The resulting sperm suspension was exposed to thermal stress at 37 °C for 5 h and analyzed hourly using CASA.

**Fig. 1:**
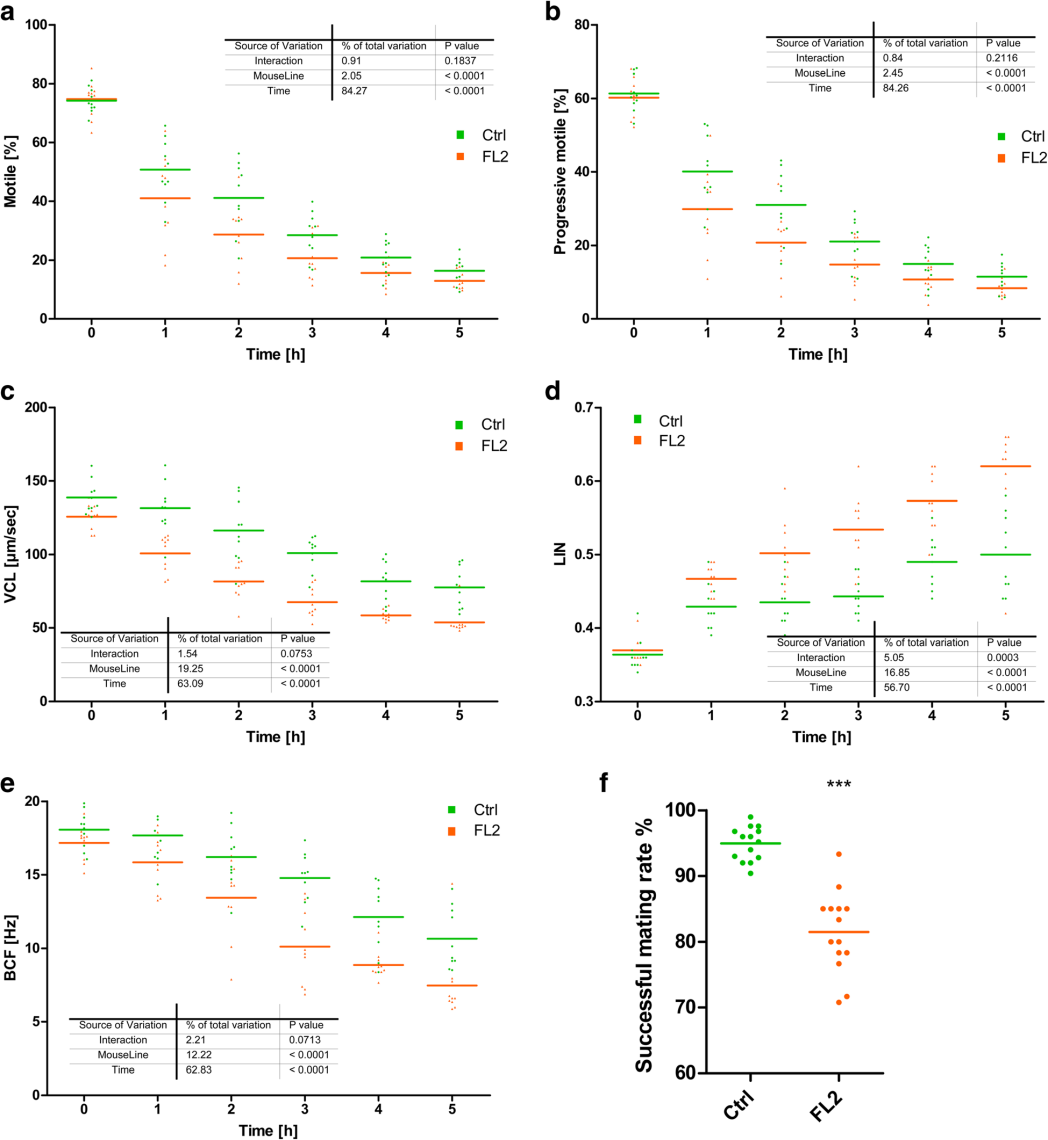
Sperm motility and birth rate. Time-course changes during thermal stress response assay of the sperm motility parameters average motility (**a**), progressive motility (**b**), curvilinear velocity (VCL) (**c**), Beat Cross Frequency (BCF) (**d**) and Linearity (LIN) (**e**) after continuing incubation at 37 °C. For this analysis, cauda epididymis spermatozoa of FL2 and Ctrl mouse line (*n* = 10 per group) were extracted and incubated at 37 °C for 5 h. Each point represents the mean of 8 defined chamber partitions viewed and measured using the Computer-assisted sperm analysis (CASA) system. The experiment has been statistically evaluated for the two factors time and mouse line effect using two-way ANOVA (GraphPad Prism) as indicated in the graph. Birth rate (**f**) of ‘high-fertility’ mouse line FL2 (orange) and Ctrl line (green) is visualized providing information about portion of successful line-specific matings. Each of the 14 points represents the average birth rate per generation. These data were acquired over a 4-year period with generation population sizes ensuring at least 60 matings. The groups were tested for normal distribution and analyzed by two-tailed t-test (***, *p* < 0.001)

The data obtained from the sperm motility experiment are shown in Fig. 1a–e. For the starting point, no significant differences in any of the motility parameters between the FL2 and Ctrl lines were observed. At 0 h, sperm motility was 74.2 ± 4.2% (SD) and 74.8 ± 6.3% for FL2 and Ctrl bucks, respectively, whereas progressively motile sperms were observed at 61.3 ± 4.9% and 60.2 ± 5.2% in FL2 and Ctrl males, respectively. As expected, the percentage of motile and progressively motile sperms rapidly decreased with thermal stress duration (see Fig. 1a & b). Notably, the sperms of FL2 animals were considerably more sensitive to thermal stress than those of Ctrl animals. Quality characteristics, such as velocity parameters (e.g., VCL Fig. 1c; VAP and VSL data not shown), revealed a significantly higher reduction for FL2 compared to the Ctrl line during the entire incubation period, most substantially within the first 60 min. Consistent with these observations, the data for linearity characteristics (e.g., LIN = VSL/VCL) showed a significant increase over the incubation period (Fig. 1d). Presumably reflecting the enhanced loss of energy necessary to ensure suitable motility, the tendency towards straightforward but slow movement increased, whereas Ctrl sperm apparently can conserve more resources for extensive, more curvilinear, oscillatory movement. Coincidently, the beat-cross frequency (BCF) as an additional indicator of oscillation behavior was significantly more diminished over thermal stress duration for FL2 than that for Ctrl (Fig. 1e).

Birth rate showed a considerable difference between the FL2 and Ctrl lines based on the evaluation of breeding information acquired over a 4-year-period and comprising at least 840 matings per group. The mating period of general maintenance breeding lasted for two weeks, enabling the occurrence of at least one ovulation. The birth rate of the Ctrl mouse line accounted for 94.9 ± 2.6% per generation, whereas the birth rate of the FL2 mouse line were registered for only 81.5 ± 6.1% (Fig. 1f). Consequently, the breeding analysis revealed a significant decrease in deliveries for the FL2 line. However, we cannot exclude this effect as accomplished by not only male but also female animals.

### Microarray analysis

Within the germinal organs, males and females share many genes essential for reproductive performance. We speculated that during selection for increased female reproductive performance, reflected in elevated ovulation rate, alterations were gradually established not only for the female but also for the male reproductive organs. To address this issue on a molecular level, we employed whole transcriptome analysis.

Testicular RNA was hybridized to individual or pooled MTA 1.0 microarrays (see microarray experimental design). The overall cell intensity distribution of each gene chip before and after normalization (SST-RMA) is depicted in the Additional file 2, illustrating an overall well-performed hybridization experiment.

**Fig. 2:**
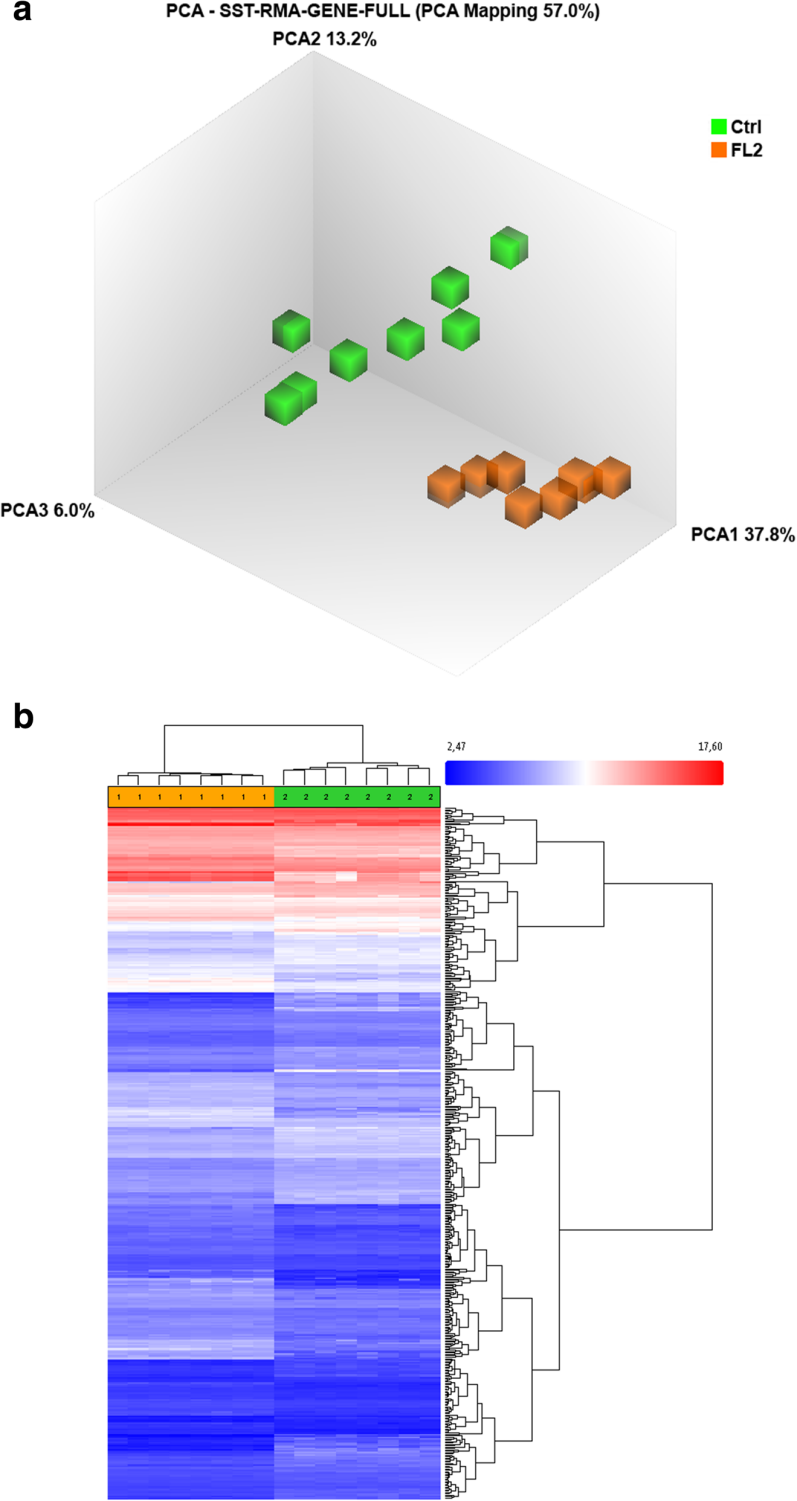
Principal component analysis (PCA) plot (**a**) of overall mRNA expression data that characterize the entire testicular transcriptional profile of enrolled samples. Each cube represents one single microarray of FL2 (orange) and Ctrl (green) mice. The PCA plot was generated using Affymetrix Expression Console software. Unsupervised hierarchical cluster analysis (**b**) of the 500 most differentially expressed genes over all groups performed. Genes are indicated vertically, the individual testicular samples are designated horizontally (FL2 orange; Ctrl green). Genes displaying upregulation are visualized in red-, genes showing downregulation are specified in blue. Intensity of the red or blue color suggests signal intensity as annotated in the scale

The Principal component analysis (PCA) was used to assess overall transcriptional differences of both mouse lines in an unbiased manner based on the first microarray experiment using testicular individual hybridization. The PCA plot illustrates a clustering according to grouping with an overall PCA mapping of 57% (see Fig. 2a). Consistent with the inbreeding coefficient (see Methods), the PCA analysis illustrated an overall more homogenous clustering of FL2 mice and the heterogenic nature of the randomly selected Ctrl line. Hence, FL2 breeding effects are visible at the overall transcriptional level prior to supervised exploration.

Hierarchical cluster analysis was based on the 500 most differentially expressed genes over all samples. FL2 and Ctrl transcripts clustered corresponding to their groups (Fig. 2b). However, we detected more homogenous gene expression within FL2 animal than in Ctrl animals.

The microarray experimental design was based on two sets of microarray experiments. For the first microarray experiment, we used 8 animals per group, whose quality-controlled testicular RNA was hybridized to individual chips. To analyze differentially expressed genes (DEGs), we applied conventional filter criteria, such as fold-change (FC) and statistical significance. Instead of only selecting a few genes for validation (via qPCR), we employed a second microarray set of biologically independent testis replicates. We used the equivalent group size of 8 animals per group. To biologically validate the DEGs of the first hybridization setup, it was sufficient to analyze the second set of independent samples as a spot test. RNA in biological replicates was pooled in equivalent amounts and suspended onto one microarray per line regarded as a verification-serving Pool-Chip. Using this approach, we ensured the comprehensive verification of the entire gene expression results obtained from the first round of microarray hybridizations.

**Fig. 3:**
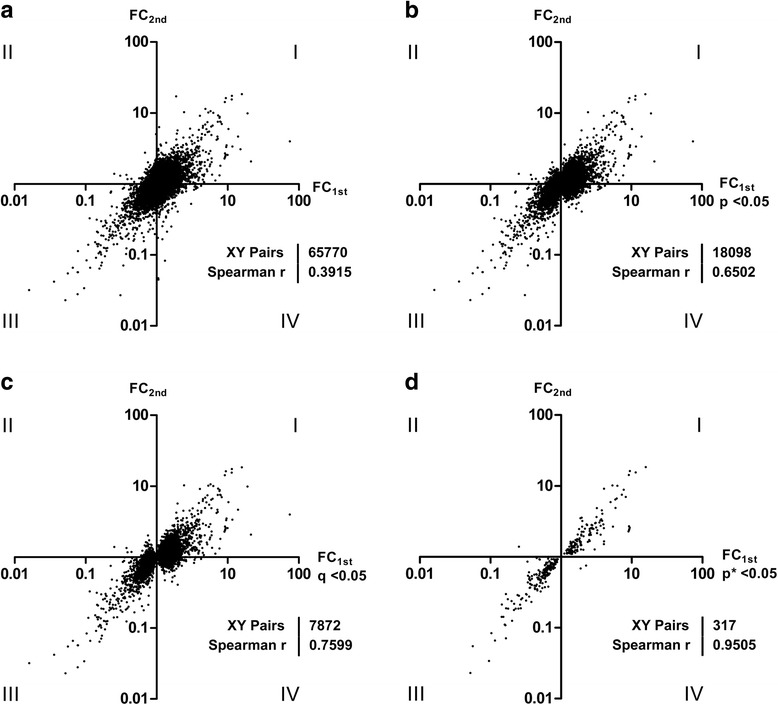
Scatter blots showing the correlation of two sets of microarray experiments. Abscissae indicate FCs of the first microarray experiment filtered statistically significant as indicated (**a**, unfiltered; **b**, ANOVA *p* < 0.05; **c**, FDR q < 0.05; **d**, Bonferroni’s correction p* < 0.05). Ordinates represent FCs of the second array validation experiment. Correlation coefficient according to Spearman (r), significance level (p) and number of enrolled XY pairs (probe set pairs) are designated

The nonparametric Spearman Correlation Analysis of these two independent microarray experiments distinctly illustrated the experimental benefit of this approach (Fig. 3). For all 65,770 PS, the correlation between FCs of the 1st and 2nd array set accounted for 0.3915 (Fig. 3a). When increasing the stringency blotting of only FCs of statistical significance in the first array set, the correlation coefficient increased from 0.6502 to 0.9505, depending on the statistical test applied (see Fig. 3b-d). Consistent with this finding, the number of probe sets passing these statistical criteria dramatically decreased from 18,098 using ANOVA *p* < 0.05 to 317 applying Bonferroni’s correction. Thus, the PS in Quadrants II and IV (Fig. 3a-d) are regarded as ‘false positive’ DEGs, reflecting a lack of biological validation of the expression intensity and direction based on the first array sets. Hence, in the case of missing information in the second microarray set, it would be impossible to filter out these ‘false positive’ genes.

**Fig. 4:**
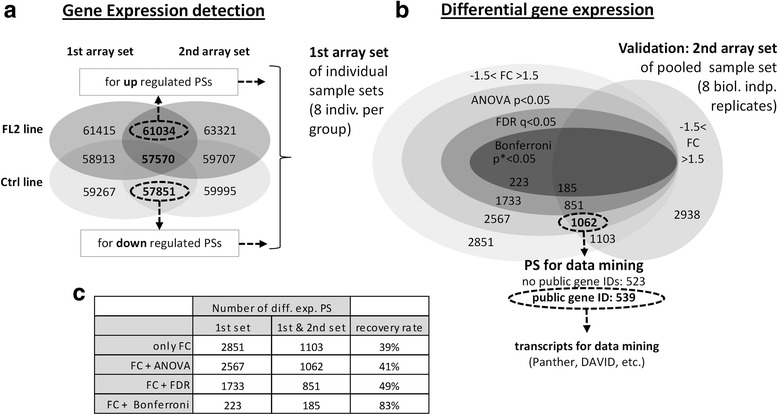
Microarray filtering strategy of differentially expressed genes (DEGs): The microarray experiment is based on two biologically independent sample sets (1st, 2nd set) of two mouse lines (FL2, Ctrl). The 1st set analyzed for differential gene expression, while the 2nd set experimentally confirmed differential expression (see section microarray experimental design Material & Methods). The gene chip MTA 1.0 (Affymetrix) is equipped with 65,770 probe sets (PS). Using the gene expression detection the number of expressed genes within each sample set was evaluated and served as first filter criteria. (**a**) The Venn diagram illustrates the number of differentially expressed genes (FL2 vs. Ctrl) detected in 1st, 2nd and both sample sets independent of statistical filter (of 1st array set ANOVA, FDR, Bonferroni). (**b**) Experimentally verified DEGs were defined as: FC > 1.5; FC < −1.5 (of 1st and 2nd set); one-way ANOVA (of 1st set). The number and percentage of DEGs of the 1st set recovered in the 2nd set are given in table (**c**)

Gene expression detection was used to filter for genes expressed in the samples. To determine whether a PS is expressed, at least 50% of the given transcript isoforms have to be detected above background. To this end, we used the expression summary based on filtering for ‘true’ or ‘false’ provided by the TCA splicing analysis algorithm. This filter of expression detection was employed in both microarray sets (see experimental design). The abundance of PS detected as present among the entire 65,770 available genes is listed for each group in Fig. 4a.

For differentially expressed genes (DEGs), we used the following filtering criteria: i) PS denoted as upregulated was expressed at least in the FL2 group; transcripts assigned as downregulated were detected at least in the Ctrl group. ii) The PS abundance was considered differentially expressed when the FC of FL2 transcripts was at least higher than 1.5 or lower than −1.5 compared to the Ctrl group in the 1st and 2nd microarray settings to be regarded as verified. Iii) The ANOVA *p*-value of the 1st array set below 0.05 was used as statistical filter.

Based on these criteria, among the 65,770 PS represented on the microarray, we detected 92% and 87% PS as expressed in FL2 and Ctrl, respectively (Fig. 4a). Only 1103 PSs passed the FC filter criterion for differential expression, for which 1061 probe sets were determined as statistically significant according to ANOVA, with a *p*-value <0.05 (Fig. 3b).

When analyzing a high number of data, correcting for the amounts of tested null hypotheses is recommended. For microarray experiments, a common algorithm is the concept of the false discovery rate (FDR). An even more stringent test is constituted by the Bonferroni’s correction. However, both methods do not reflect the biological diversity. Thus, we used ANOVA in combination with the experimental validation method. Using biologically independent sample sets, we observed only moderate ‘recovery’ rates of 48%, 54% or 84% using ANOVA, FDR or Bonferroni’s correction as a filter, respectively (Fig. 4c). Although a robust statistical filter ensures an increased prediction rate in unknown sample sets, as in the 2nd microarray set, the number of faithfully positive PS was only 48% to 84%, depending on the statistic filter used. Using a more stringent statistical filter would only reduce the number of DEGs, i.e., when applying Bonferroni’s correction.

**Fig. 5:**
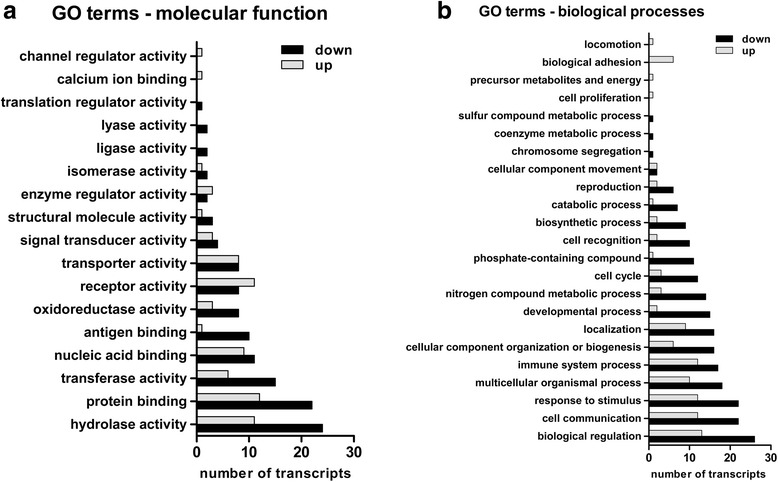
Gene ontology (GO): for up (gray) and down (black) regulated transcripts annotated for molecular functions (**a**) and biological processes (**b**)

Gene ontology (GO) classification was performed for all differentially expressed transcripts filtered by our double-array-set validation. Among the 1061 differentially expressed PSs, almost 50% (523 PS) of these genes could not be annotated with official gene symbols or public gene IDs, reflecting the missing database knowledge of these mainly noncoding transcripts (Fig. 4b). Hence, we excluded these transcripts from functional interpretation processing. A list of the DEGs is provided in Additional file 3. GO classification identified hydrolase activity, protein binding and transferase activity as major molecular functional groups (geneontology.org) [9, 10]. However, within these three classifications, more than twice as many transcripts were downregulated than upregulated, indicating reduced activity at the transcriptional level in the FL2 group (Fig. 5a). Similar results were obtained when classifying the biological processes (Fig. 5b). Among these broad biological annotations, the number of downregulated transcripts also exceeded the amount of upregulated DEGs. However, we observed that this GO alignment lacks almost 50% of the 539 applied transcripts, reflecting missing annotations within the database geneontology.org.

Cluster analysis was performed to investigate whether particular chromosomal regions are differentially expressed, potentially resulting from the breeding process. In general, particular genes of close relations can be organized into clusters within the eukaryotic genome. Such clusters are up to 300 kb in size [15–17]. Hence, we explored whether particular DEGs, showing similar expression, map to closely related genomic regions.

For example, seventeen transcripts of the immunoglobulin heavy chain (J558 family) cluster located on Chr12 62.59 cM are less active in FL2 than in Ctrl mice. However, it cannot be excluded that there might be an indirect effect resulting from a reduction in diversity and the breeding process and the genetic separation for more than 170 generations causing an undirected allelic drift rather than an active regulation. In addition, Chr9, 1.83–1.84 cM, harbors a region of 8 consecutive upregulated (FC 2.0–9.9) genes. These genes are encode transcripts of unknown function.

An additional locus of differential expression is the kallikrein-related peptidases (KLKs) cluster. This cluster is located on Chr7, 28.26–28.74 cM. Although the KLKs cluster comprises 26 KLKs, only 14 of these enzymes are downregulated Additional file 4. The subset of differentially expressed KLKs is expressed within the testis, whereas KLKs are generally unexpressed in the testis and are not actually detected as differentially expressed within the testis transcriptomics data. Thus, these findings suggest an apparent active regulation of these differentially expressed KLKs rather than decreased activity of the whole KLK cluster.

Gene set enrichment analysis (GSEA) was used to evaluate functional differences between the FL2 and Ctrl testicular transcriptomes. A total of 539 DEGs with official geneIDs were subjected to Database for Annotation, Visualization and Integrated Discovery (DAVID). Among the Gene ID transcript list, 477 genes matched to murine annotations within the GO database. However, when performing the GSEA, only 50% of these IDs could be assigned to functional annotations in DAVID. A subset of the main enrichment functions and corresponding genes is presented in Table 4.

The bioinformatics GSEA disclosed a wide range of biological and molecular categories obviously affected in the testis of FL2 bucks during long-term breeding. The most prominently affected genes are those of the renin-angiotensin system, with a fold-enrichment of 27.5. Most of the DEGs within this category are constituted by serine proteases. Indeed, molecular functional classification on the one hand unveiled an overrepresentation of transcripts associated with serine-type endopeptidase activity (fold enrichment 6.7), most of which were located within the so-called KLK cluster. On the other hand, we detected genes with serine-type endopeptidase inhibitor activity (fold-enrichment 4.3). In addition to the peptides involved in proteolytic processes, the genes of the steroid hormone biosynthesis cascade were also observed (fold-enrichment 4.4), and most of these genes were associated with ovarian steroidogenesis (fold-enrichment 6.8). Moreover, Jak-STAT signaling pathway transcripts represented a third broad category of differentially expressed genes within the FL2 transcriptome (fold-enrichment 3.3), a majority of which were associated with cyclin-dependent protein serine/threonine kinase activity (fold-enrichment 9.8).

Mammalian phenotypes (MP) Based on the overall assumption to detect selection-induced alterations in testicular reproduction gene expression, we searched the DEG lists for genes associated with reproductive phenotypes within the database Mouse Genome Informatics (MGI - www.informatics.jax.org). We expected genes associated with male and female fecundity phenotypes. Among more than 2000 genotypes associated with reproductive phenotypes, we detected 17 differentially expressed genes in the FL2 mouse line. These genes are summarized in Table 3, with a selection of the most pronounced reproductive phenotypes. This list also included the genes Gdf9 and Agt, which are important for proper ovulation. Both genes were upregulated in FL2 testis. In contrast, Cyp19a1, a gene associated with asthenozoospermia, oligozoospermia and female fecundity, was less expressed in FL2 bucks.

**Table 3:** List of genes associated with reproductive phenotypes of the database Mouse Genome Informatics (MGI - www.informatics.jax.org) mapping to DEGs. Genes and the corresponding mammalian phenotypes (MP) are indicated

Gene Symbol	FC 1st set	FC 2nd set	Term	MP ID
Abca1	−3.2***	−2.8	Male infertility	MP:0001925
			Female infertility	MP:0001926
			Abnormal ovary morphology	MP:0001126
			Abnormal testis morphology	MP:0001146
Ctsh	−3.1***	−3.4	Reproductive system phenotype	MP:0005389
Gdf9	−3.0***	−2.3	Female infertility	MP:0001926
			Abnormal oogenesis	MP:0001931
			Abnormal ovarian folliculogenesis	MP:0001130
			Increased circulating fsh	MP:0001750
			Increased circulating lh	MP:0001751
Agt	−2.8***	−2.1	Decreased ovulation rate	MP:0003355
Gpr133/Adgrd1	−2.8**	−3.0	Female infertility	MP:0001926
Eno4	−2.8***	−2.4	Male infertility	MP:0001925
			Asthenozoospermia	MP:0002675
			Oligozoospermia	MP:0002687
Vcam1	−2.0***	−2.2	Reproductive system phenotype	MP:0005389
Pttg1	−1.7***	−1.8	Reduced fertility	MP:0001921
			Decreased litter size	MP:0001935
			Decreased testis weight	MP:0004852
Hoxa5	−1.6*	−1.6	Abnormal estrous cycle	MP:0001927
Nr2c2	−1.5**	−1.5	Reduced male fertility	MP:0001922
			Oligozoospermia	MP:0002687
Cyp19a1	1.7***	1.7	Male infertility	MP:0001925
			Female infertility	MP:0001926
			Impaired ovarian folliculogenesis	MP:0001129
			Asthenozoospermia	MP:0002675
			Oligozoospermia	MP:0002687
			Leydig cell hyperplasia	MP:0001152
			Increased circulating fsh	MP:0001750
			Increased circulating lh	MP:0001751
Adamts5	1.8***	1.7	Reproductive system phenotype	MP:0005389
Atxn7	1.8***	1.8	Male infertility	MP:0001925
			Female infertility	MP:0001926
Clec4a2	2.0***	1.6	Seminiferous tubule degeneration	MP:0001154
Antxr2	2.6**	2.6	Female infertility	MP:0001926
Ccnd2	3.1***	3.0	Abnormal ovulation	MP:0001928
			Abnormal ovarian folliculogenesis	MP:0001130
			Oligozoospermia	MP:0002687

FC of 1st (*, *p* < 0 .05; **, *p* < 0 .01; ***, *p* < 0 .001) and 2nd microarray set are provided in the table

### Validation of microarray data using RT-qPCR

**Fig. 6:**
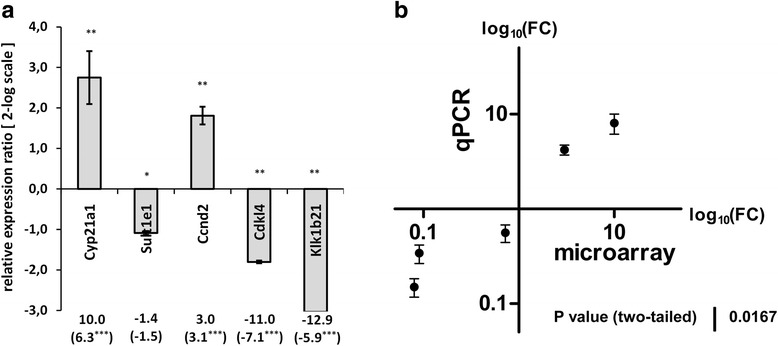
Real-time qPCR. Validation of the 2nd set of microarrays to verify the pooling approach using quantitative real time PCR as independent method. RNA of individual FL2 and Ctrl mice was used prior of pooling for the microarray. Samples were normalized to a combination of reference genes (36B4, GAPDH, HPRT, B2m) and statistically evaluated employing the Relative Expression Software Tool (REST 2009) [12]. Relative expression ratio are presented as log2 bars (**a**) (* *p* < 0.05; *** *p* < 0.001). Numbers below bars indicate fold change (FC) values calculated for the 2nd set of microarrays comprising the same animals as pooled samples. Numbers in brackets are FCs of the 1st set of microarrays as biologically independent replicates. Scatter blot illustrates the FCs acquired by both microarray (abscissae) and qPCR (ordinates), based on the same set of animals (**b**)

A select group of DEGs were re-analyzed using quantitative real-time PCR. The intention of this re-evaluation was to confirm the overall experimental pooling approach of the 2nd set of microarrays. Based only on equal amounts of pooled samples, this 2nd set missed individual variation. Hence, we used the same isolated RNA samples of the 2nd microarray set prior to pooling and analyzed the relative transcriptional abundance of each sample individually via qPCR. Both methods differed in the detection target. Microarray data are based on the binding of 20-bp probes distributed from 5′ to 3′. In contrast, the amplification of a 150–250-bp amplicons is the basis of real-time PCR. Although traces of genomic DNA were removed in the RNA extraction process, the primers were designed as oligonucleotides, particularly sequences binding to intron-overspanning exons, when possible. The overall result of the qPCR suitably demonstrated a correlation with the microarray data (Fig. 6b), thus also experimentally confirming both methods and evaluations as based on the same sample set of RNAs.

The analyzed transcripts were significantly expressed regarding Cyp21a1 (FC 8.1; *p* < 0.01), Sult1e1 (FC −1.8; *p* = 0.011), Ccnd2 (FC 4.2; *p* < 0.01), Cdkl4 (FC −2.9; *p* < 0.01) and Klk1b21 (FC −6.8; *p* < 0.01). The FCs and the *p*-value of the first microarray experiment exhibited significant similarity to the biologically independent replicates (2nd set) (see Fig. 6a, number in brackets). Consequently, the comparison convincingly verified the suitability of the applied microarray experimental design.

### Progesterone concentration in blood

Among the most differentially expressed genes of the microarray results, Cyp21a1 was also detected (FC: 6.3***, 10.0; 1st and 2nd array set, respectively). This gene encodes the enzyme steroid 21-hydroxylase, which is essential for converting progesterone (P4) to 11-deoxycorticosterone. Although this enzyme is generally associated with adrenal gland activity, there have also been reports of peripheral expression. Indeed, the average signal intensity of the microarray experiment was clearly above background (Ctrl: Avg Signal (log2): 5.84, 5.41 (1st, 2nd set); (FL2: Avg Signal (log2): 8.48, 8.73 (1st, 2nd set)).

To analyze whether this differential expression of Cyp21a1 results from hormonal alterations, the serum progesterone concentration of the males was examined. To minimize the influence of circadian fluctuation, all serum samples were collected at the same time of day.

**Fig. 7:**
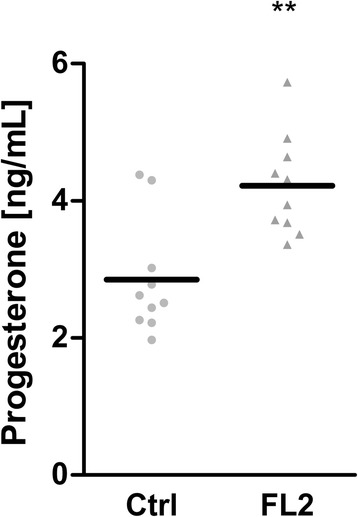
Progesterone. Plasma progesterone concentrations of FL2 and Ctrl males were analyzed by RIA. Individual and mean levels are indicated. Groups were tested for normal distribution and analyzed by two-tailed t-test (**, *p* < 0.01)

In Ctrl males, we measured 2.9 ± 0.8 ng/ml total P4 from blood plasma samples (Fig. 7). This concentration was slightly higher compared with C57BL/6 male mice [18]. In contrast, bucks of the FL2 showed significantly increased P4 values of 4.2 ± 0.7 ng/ml (*p* < 0.001). We previously determined serum corticosterone values for FL2 by GC/MS [6]. Corticosterone acts downstream of P4 in the corticosteroid-aldosterone metabolic pathway and hence could be induced by enhanced P4. However, these corticosterone differences did not pass statistical significance (FL2: 30.8 ± ng/ml; Ctrl: 26.7 ± ng/ml).

## Discussion

Research on reproductive phenotypes primarily focuses on animal models of reduced fertility. The majority of these models are based on transgenic or knockout approaches. Hence, currently, little is known about increased fertility and the gender specific molecular alteration of this phenotype. In the present study, we investigated whether and to what extent male animals are affected by long-term selected breeding for ‘high’ reproductive performance of exclusively female focused fecundity traits.

The study object was a worldwide unique mouse fertility line (FL2) generally distinguished by almost doubling number of pups per litter and total litter weight (Tab. 2), obviously reflecting increased ovulation rate, as previously described [4]. To further previously accomplished initial characterization studies founded on notable changes in endocrinology and behavior, the aim of the present study was to shed light on physiological traits, such as sperm motility and transcriptome alterations, which might have been generated on the male side in response to more than 170 generations of female focused selected breeding.

Sperm motility and overall functional sperms are the souls of male reproduction. Therefore, we examined sperm motility as a physiological characteristic. To this end, FL2 and Ctrl spermatozoa were released out of the cauda epididymis and subsequently exposed to 37 °C thermal stress for 5 h and hourly evaluated for several motility characteristics based on CASA system measurements. Initial assessment immediately after preparation (0 h measurement) showed no substantial difference among FL2 and Ctrl bucks with acquired data equivalent to the literature [19]. However, surprisingly, thermal exposure apparently prompted a considerable reduction in overall motility performance for FL2 spermatozoa. The amount of motile and progressively motile sperm was significantly decreased during 5 h thermal stress and substantial diminishing of entire quality parameters, such as speed of movement (e.g., VCL), Linearity (e.g., LIN) and oscillation (e.g., BCF). Hence, these findings suggest that FL2 sperm substantially suffer from temperature increases of approximately 3 °C higher than the physiological environment (34–35 °C). Thus, initially after exposure, FL2 spermatozoa begin to extensively lose motility capability continuing in minimized motility fractions over the whole experimental period. Spermatozoa able to persist viability are distinguished by speed impairments, which lead to short distances passed compared to Ctrl. Furthermore, consistent with prolonged thermal stress, FL2 spermatozoa showed to increasing loss in the maintenance of curvilinear and oscillatory paths during progression. Apparently under stress, the suitable metabolic activity required for vital motility associated with progressive, fast and wriggled, oscillatory movement can no longer be provided. Loss of granted energy leads to essentially slower and more straightforward FL2 spermatozoa movement. Consequently, the detection of enhanced linearity and oscillation parameters for FL2, such as LIN and BCF, during the thermal stress period is presumably a reflection of comprehensive spermatozoa damage. Furthermore, P4 triggers the hyperactivation of spermatozoa. FL2 females and males exhibit an increase in P4 level [4], see Fig. 7. These elevated P4 levels could reflect the selection for increased litter size to support larger pregnancies. Thus, FL2 spermatozoa might be adapted to high-progesterone intrauterine environments. FL2 sperms are more susceptible to P4 loss, as observed in M199 media, which was used for the CASA experiments in the present study. Hence, the observed decrease of FL2 sperm motility performance alleviated curvilinear velocity and increased linearity.

Thus, the causes of FL2 sperm motility reduction with respect to thermal noxa remain unknown, and clarification of the potential essential restrains in FL2 spermatozoa performance across the entire fertilization process remain elusive. For example, not only metabolic activity but also sperm capacitation and oocyte fertilization performance in single and competitive conditions should be assessed. These future assays together with the findings of the present sperm motility studies will hopefully lead to profound FL2 sperm characterization necessary to generalize FL2 sperm as substantially worse in performance compared to unselected Ctrl lines.

Birth rate breeding data were obtained during a 4-year period. However, these physiological data show a considerable reduction of birth rate for the FL2 line. These data are based on at least 60 breeding pairs per generation, reflecting the overall reduced delivery rate over 14 generations. In addition to these data, we collected data from two-factorial breeding experiments crossing males and females of the Ctrl and the FL2 line in all combinations. Within this setting, we registered no significant differences in any of the groupings [6]. However, admittedly, the group size of this experiment was considerably less than the obtained breeding values over 4 years.

Furthermore, these findings do not exclusively imply that FL2 males contribute to alleviating mating success. However, previous data indicate no reduced female fecundity performance. For example, FL2 females ovulate approximately 24 oocytes. Moreover, based on in vivo studies, almost every FL2 oocyte was fertilized and developed to an embryo [4]. Taken together, these results may explain the weaker FL2 birth rate resulting from the male gender. Nevertheless, additional in vitro fertilization studies are needed in the future.

### Transcriptional alterations (genes, phenotype, cluster)

The comparative whole transcriptome expression profile was used to identify testicular genes whose expression pattern has been potentially affected by the long-term selection for increased female reproductive performance. Thus, we expected some of detected differentially expressed genes (DEGs) to be directly causative of the female focused breeding process. Other genes might be regulated as ‘secondary’ effect within the testis. However, in both cases, transcriptional alterations might actually contribute to the FL2 testicular phenotype. Hence, we discussed DEGs in light of male and female reproduction.

Functional annotation clustering revealed that most enrichment scores for the renin-angiotensinogen system (RAS) and for a set of RAS genes encoding serine-type endoprotease activity. RAS is a hormonal system for blood pressure regulation [20]. In addition, there is increasing evidence of the local synthesis of RAS components in various tissues [21]. A majority of differentially expressed RAS-associated genes in FL2 males mapped to tissue kallikrein-related peptidases (KLK). These KLKs are organized in tandem within a cluster on Chr7 [22]. In FL2, most of these differentially expressed KLK transcripts mapped to genes expressed in the murine testis. Other KLK cluster genes not expressed in the testis were indeed not altered within this dataset compared to the Ctrl line. This tissue-specific effect suggests the active regulation rather than decreased activity of the entire KLK cluster locus (Additional file 4).

The physiological function of the kallikrein system within the testis has been only partly elucidated. Using the term KLK in combination with testis, eight publications in the PubMed database were detected. Particularly, KLKs are discussed as prognostic tumor markers [23]. However, the beneficial effect on asthenozoospermia and oligozoospermia of systemic kallikrein administration has been recognized in clinical interventional studies [24, 25]. Consequently, the downregulation of KLKs might potentially explain the decreased sperm motility parameters observed in FL2 bucks.

In addition to the local function of KLKs in the testis [26, 27], there have been reports of kallikrein − kinin systems (KKS) interacting in RAS [28–30]. In fact, the differential expression of KLK genes was accompanied by alterations in the angiotensinogen (Agt) transcriptional level. Agt is one of the major players in the RAS, acting as a precursor of the angiotensin cascade. There is evidence for the direct interaction of kallikrein on the generation of angiotensin (Ang) II in male reproductive organs rather than the ‘canonical’ enzyme cascade via renin [31]. Nevertheless, the role for angiotensin effectors in the testis is only partially understood.

In addition to its implication in male reproduction, RAS and KLKs are of vital importance for ovarian function, including the systemic action of RAS on blood pressure control and vascular function. There is also evidence for local tissue events of RAS components [32, 33]. Thus, the dysregulation of the Agt gene in transgenic females decreases the ovulation rate [34]. In addition, antagonizing Ang II type 1 receptor (AGTR1) in dominant follicles blocks follicle growth and rupture and decreases Cyp19a1 and Ccnd2 mRNA expression. Surprisingly, the opposite pattern was observed in FL2 testis. The Agt transcript level was decreased, while Cyp19a1 and Ccnd2 transcripts were elevated. Furthermore, the perfusion of Ang II in rat ovaries induces progesterone (P4) synthesis. Consistently, we detected enhanced serum P4 concentrations in FL2 males.

Ccnd2 is important in the testicular and ovarian cell cycles [35, 36]. Genetic intervention leads to oligozoospermia and abnormal ovarian folliculogenesis [37]. The Ccnd2 gene is induced by FSH and regulates cell proliferation in the gonads [37]. Downregulation by dihydrotestosterone (DHT) induces cell cycle arrest in granulosa cells [38]. Moreover, D-type cyclins can be regulated via the intracellular Jak/STAT signaling pathway [39–41]. In the present study, functional annotation clustering indicated the enrichment of Jak/STAT pathway-associated genes in FL2 testis (see Table 4 for dedicated genes). This pathway has been implicated in the regulation of cell cycle progression mediating extracellular signaling. Within FL2 testis Jak/Stat dependent receptors (LIFR, CNTFR, Il3ra) and SOS1 are repressed, whereas intracellular Ccnd2 is upregulated. LIF and CNTF signaling acts on testis and ovarian functions [42–46]. Furthermore, we detected the decreased expression of cyclin-dependent kinase (Cdk) Cdkl4, Cdk14 and Cdk15 in FL2 testis compared to Ctrl. Although the action of cyclin/cdk complexes on cell cycle progression is widely known [47, 48], the interaction of these particular genes within male or female germinal organs remains unknown [49]. However, studies have shown that Jak/STAT is associated with homeostasis of stem cell niche within Drosophila testis and ovaries [50].

**Table 4:** Gene Ontology (GO) term enrichment analysis for testis of FL2 versus Ctrl line using the platform Database for Annotation, Visualization and Integrated Discovery (DAVID). Functional Annotation Clustering of differentially expressed genes in FL2 versus Ctrl testis. The analysis for the top overrepresented GO terms with fold-enrichment scores and Benjamin’s correction are indicated

Term	Genes	Fold Enrichment	Benjamini
Renin-angiotensin system	AGT, KLK1B21, KLK1B8, KLK1B22, KLK1B9, KLK1B11, KLK1B26, KLK1B24, KLK1B1, MAS1	27.5	6 × 10^−09^
Serine-type endopeptidase activity	KLK1B21, KLK1B8, KLK1B22, KLK1B9, KLK1B11, KLK1B26, KLK1B4, KLK1B24, KLK1B1, EGFBP2, KLK1B16, KLK1B27, CTSH	6.7	9 × 10^−05^
Serine-type endopeptidase inhibitor activity	SERPINE3, SERPINA3G, AGT, SERPINA3C, SERPINA3A	4.3	8 × 10^−01^
Ovarian steroidogenesis	CYP1B1, HSD3B6, GDF9, CYP19A1	6.76	6 × 10^−01^
Steroid hormone biosynthesis	CYP1B1, HSD3B6, CYP21A1, CYP19A1	4.43	7 × 10^−01^
Fatty acid biosynthetic process	ELOVL3, AGMO, TECR, OXSM, NDUFS6	5.77	9 × 10^−01^
Jak-STAT signaling pathway	CCND2, SOS1, LIFR, CNTFR, IL3RA	3.32	7 × 10^−01^
Cyclin-dependent protein serine/threonine kinase activity	CDK15, CDK14, CDKL4	9.81	9 × 10^−01^

Moreover, a group of differentially expressed genes mapped to the steroid hormone synthesis and ovarian steroidogenesis. Steroids are of vital importance for male and female reproduction regulation. The Abca1 transcript is involved in cholesterol trafficking. Consequently, transgenic intervention leads to impaired cholesterol levels [51, 52], resulting in male and female infertility paired with abnormal reproductive organ morphology in mice [53–55]. This rate-limiting gene was decreased in FL2 testis. Further, Cyp19a1, the gene encoding aromatase, which is essential for converting testosterone to estradiol (E2), was upregulated in FL2. This gene has been targeted by several transgenic interventions leading to phenotypic alterations, such as impaired spermatogenesis and ovarian folliculogenesis, manifested in the reduced fertility of both sexes [56–59]. Interestingly, the expression level of Sult1e1 was also diminished in FL2 testis compared to Ctrl. E2 is inactivated by the steroid sulfatase Sult1e1, commonly regarded as the only relevant sulfotransferase for estrogens [60]. The enzyme is present in the ovary, whereas expression and testicular function have not been reported [61]. However, sulfated steroid inactivation and transport and local storage of steroids have been postulated [60]. Moreover, the overexpression of Sult1e1 counteracts the estrogen-mediated proliferation and decreased expression of D-cyclin [62]. Indeed, the level of Ccnd2 was elevated, while Sult1e1 mRNA was decreased in FL2 testis compared to Ctrl.

Cyp1b1 expression was decreased in FL2 males. This enzyme catalyzes several reactions among the hydroxylation of estradiol for the elimination process [63]. Although Cyp1b1 has been genetically targeted, information for its effects on the reproductive system is still lacking [64, 65]. However, polymorphisms in Cyp1b1 may augment the risk of abnormal sperm parameters [66].

A key enzyme in the biosynthesis of steroids is 3β-hydroxysteroid dehydrogenase, which is essential for the production of progesterone (P4) and functions as a precursor for the synthesis of androgens, estrogens, glucocorticoids and mineralocorticoids [67]. In this experimental setting, the Hsd3b6 transcript was downregulated in FL2 testis compared to Ctrl. In mice, the isoform type VI (Hsd3b6) is expressed in the testis from puberty to adulthood in interstitial Leydig cells as and placental giant trophoblast cells, potentially suggesting embryonic-placental implantation [67, 68]. Decreased Hsd3b6 levels, as observed in FL2, have been associated with adverse male reproductive function in several studies [69–71], however we currently lack genetic interventional studies clearly dissecting its functional role in reproduction.

Furthermore, Cyp21a1 was considerably upregulated. This enzyme acts downstream of P4, converting the steroid to 11-deoxycorticosterone. To examine the differential mRNA expression of both enzymes, we analyzed the level of P4 in serum using RIA. P4 concentrations in Ctrl males were the same magnitude as previously published [18, 72]. Interestingly, the P4 levels of FL2 bucks were 50% elevated. Increased P4 levels have previously been observed in FL2 females compared to the Ctrl line [4]. In addition, FL2 females have more CLs [4]. However, no correlation between CLs and P4 levels has been reported [4]. For males, we currently have no indication for elevated steroidogenic cell numbers in FL2 testis (data not shown), suggesting the ‘active’ regulation of high P4 levels.

In terms of considering an integrating picture, some of the effects observed in FL2 males were consistent with the work of Harini et al., showing the effects of prenatal exposure to progesterone on male reproductive parameters at adulthood [73]. After birth, the females received three injections of P4 over pregnancy (7 mg P4/kg), adopting human interventional studies to mouse species. Mice of the resulting F1 generation were paired with normal cycling females for recording of reproduction traits. Intriguingly fetal exposure to high P4 levels apparently resulted in several adverse reproductive effects, e.g., decreased motile and viable sperms, low levels of Hsd3b activity and reduced fertility index, consistent with the observed effects on birth rate.

Although P4 is a ‘female hormone’, serum P4 levels in males do not differ from female levels out of luteal phase [74, 75] or in post-menopausal women [76, 77]. In men, P4 has been associated with processes of spermatogenesis and hence functional loss will lead to infertility [78, 79]. P4 triggers hyperactivation in spermatozoa, leading to a rapid increase in Ca^2+^, which is potentially mediated by the GABA_A_ receptor [80]. This hormone can also serve as a precursor for corticosterone to feed the corticosteroid/aldosterone pathway. We previously analyzed serum corticosterone levels in males by GC/MS [6]. Although corticosterone levels are slightly elevated in FL2 compared to Ctrl males, these differences were not statistically significant. In males, this hormone is either testicular or of adrenal origin. However, P4 is known as a hormone for female reproduction and ovulation. Other approaches to obtain an integrative perspective of these FL2 transcriptomics data should consider the observed differential KLK expression: It is known that progesterins and other steroids control the expression of multiple kallikreins [81, 82]. For example, epostane, representing an antagonist of 3β-hydroxysteroid dehydrogenase (HSD3), can inhibit ovulation while exogenous P4 induces kallikrein and reverses the anovulatory effect of the antagonist [83]. Nevertheless, steroids induce KLK expression, whereas we detected the decreased expression of several KLKs.

## Conclusion

To our knowledge, the present study is the first report depicting an in-depth transcriptomics analysis of high-fertility male mice with female-focused breeding on augmented reproductive performance compared to a still existing ‘founder’ population of an unselected control line.

In combination with additional physiological phenotype studies, we showed that long-term selection accompanied by an increased ovulation rate [4] in females also affects the testicular gene expression of transcripts associated with female fecundity and ovulation. In addition, decreased sperm motility parameters for FL2 bucks could be revealed when semen is exposed to stress response analyses. Furthermore, the FL2 line displayed a markedly alleviated birth rate in long-term breeding assessment studies. However, based on the birth rate data obtained from standard breeding values in the present study, we cannot differentiate to what extent the FL2 gender is responsible for the decreased mating rate. Hence, the results of the present study should be considered as a starting point to shed light on the definite transcriptomics and genotypic alterations distinctly manifested on male side during 40 years of murine outbred breeding towards high-fertility phenotypes.

## Additional files

Additional file 1: List of RT-qPCR primers used. (DOCX 52 kb)

Additional file 2: Distribution of probe cell intensity of unprocessed raw data (.CEL data) (a) Distribution of signal intensity values (.CHP data) after normalization by the Robust Multiarray Average with Signal Space Transformation algorithm (SST-RMA). (b) Consideration of both plots implies an overall successful hybridization experiment for all processed MTA 1.0 microarrays. (ZIP 63 kb)

Additional file 3: List (Excel file) of differential expressed transcripts (FL2 vs. Ctrl). DEGs were defined as: FC > 1.5; FC < −1.5 (of 1st and 2nd set); one-way ANOVA (of 1st set). (XLS 988 kb)

Additional file 4: Kallikrein (KLK)-Cluster within the murine genome is located on Chromosome 7 and spans from 28.26 to 28.74 cM. The orientation and organization within the cluster is illustrated. Color intensity of up (red) and down (green) regulated gene expression in FL2 testis in relation to the Ctrl group is depicted. (TIFF 766 kb)

## Abbreviations

BCF: Beat cross frequency
CASA: Computer- assisted sperm analysis
CL: Corpus luteum
Ctrl: Control mouse line
DABG: Detection above background
DEG: Differential expressed genes
E2: Estradiol
EC: Expression console software
FC: Fold-change
FDR: False discovery rate
FL2: Fertility mouse line 2
GO: Gene ontology
KKS: Kallikrein − kinin systems
KLK: Kallikrein-related peptidases
LIN: Linearity
P4: Progesterone
PCA: Principal component analysis
PS: Probe set
RAS: Renin-angiotensinogen system
RMA: Robust multiarray average (algorithm)
SST: Signal space transformation
TAC: Transcriptome analysis console software
VCL: Curvilinear velocity
