# Supplementary material for: Selection for female traits of high fertility affects male reproductive performance and alters the testicular transcriptional profile
Source: BMC Genomics. 2017 Nov 21;18:889. doi: 10.1186/s12864-017-4288-z (PMC5697431; doi:10.1186/s12864-017-4288-z)
Supplement: Supplementary file 1 — List of RT-qPCR primers used. (DOCX 52 kb) [file 12864_2017_4288_MOESM1_ESM.docx]

***Supplementary 3:*** *List of used primers for RT-qPCR.*

| Gene Symbol | Gene ID  NCBI | Sequence | Product [bp] |
| --- | --- | --- | --- |
| *Cyp21a1* | 13079 | fw – CTCCTGCCTCACTTTTGGAG  re – TATGGTCCCGACTCTCTTGG | 185 |
| *Sult1e1* | 20860 | fw – gTggAAAAATgCAAggAggA  re – AAgAAACggCgACATCTTTg | 217 |
| *Ccnd2* | 12444 | fw – CTgCggAAAAgCTgTgCATT  re – AACTTgAAgTCggTAgCgCA | 226 |
| *Cdkl4* | 381113 | fw – ACCTgAACCAgAggACATgg  re – CCAggAATAAgAggCAgCAg | 237 |
| *Klk1b21* | 16616 | fw – TTGTTGGAGGATTTAACTGTGAGA  re – AGGATTCATGTTGGAATAGCTTGT | 183 |
| *36B4* (Ref.) | 11837 | fw – TAACCCTgAAgTgCTCgACATC  re – ggAAggTgTACTCAgTCTCCAC | 182 |
| *GAPDH* (Ref.) | 14433 | fw – TCACCATCTTCCAggAgCgAgAC  re – TTTCTCgTggTTCACACCCATCA | 197 |
| *B2m* (Ref.) | 12010 | fw – TGTCTCACTGACCGGCCTGTATGCT  re – CAGTGGGGGTGAATTCAGTGTGAGC | 245 |
| *HPRT* (Ref.) | 15452 | fw – GCTTGCTGGTGAAAAGGACCTCTCG  re – TTTGCAGATTCAACTTGCGCTCATC | 198 |
