# Supplementary figures and images for: Selection for female traits of high fertility affects male reproductive performance and alters the testicular transcriptional profile
Source: BMC Genomics. 2017 Nov 21;18:889. doi: 10.1186/s12864-017-4288-z (PMC5697431; doi:10.1186/s12864-017-4288-z)

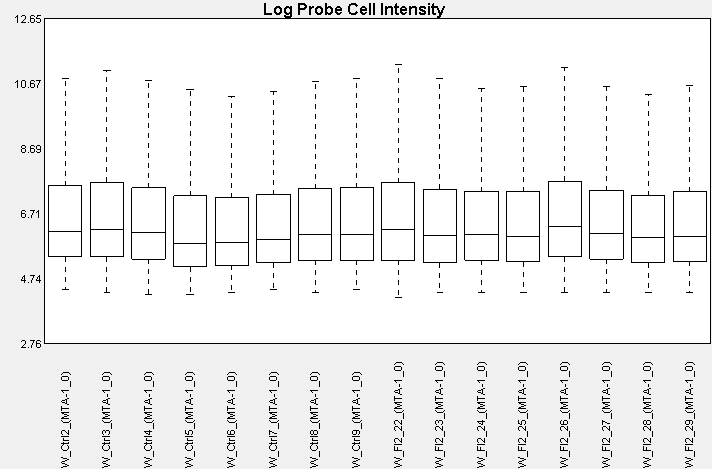

Supplement: Supplementary file 2 — Distribution of probe cell intensity of unprocessed raw data (.CEL data) (a) Distribution of signal intensity values (.CHP data) after normalization by the Robust Multiarray Average with Signal Space Transformation algorithm (SST-RMA). (b) Consideration of both plots implies an overall successful hybridization experiment for all processed MTA 1.0 microarrays. (ZIP 63 kb) [file 12864_2017_4288_MOESM2_ESM.zip › Supplementary1a.tif]

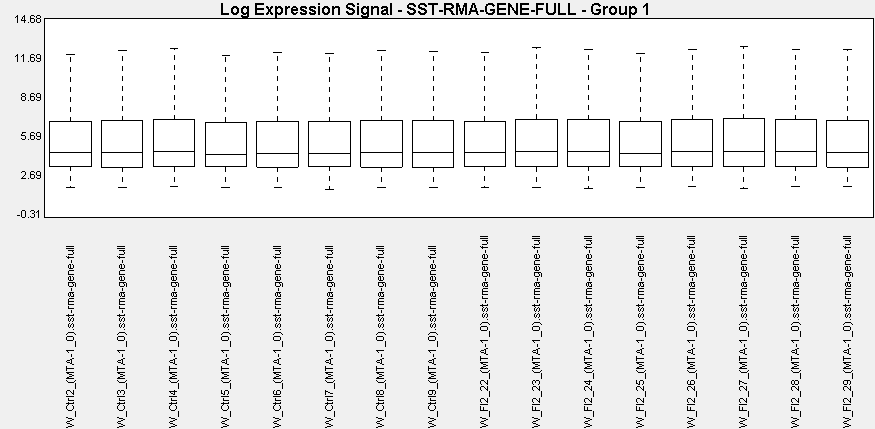

Supplement: Supplementary file 2 — Distribution of probe cell intensity of unprocessed raw data (.CEL data) (a) Distribution of signal intensity values (.CHP data) after normalization by the Robust Multiarray Average with Signal Space Transformation algorithm (SST-RMA). (b) Consideration of both plots implies an overall successful hybridization experiment for all processed MTA 1.0 microarrays. (ZIP 63 kb) [file 12864_2017_4288_MOESM2_ESM.zip › Supplementary1b.tif]
